# Dissecting the Genetic Architecture of Morphological Traits in Sunflower (*Helianthus annuus* L.)

**DOI:** 10.3390/genes15070950

**Published:** 2024-07-19

**Authors:** Yavuz Delen, Semra Palali-Delen, Gen Xu, Mohamed Neji, Jinliang Yang, Ismail Dweikat

**Affiliations:** 1 Department of Agronomy and Horticulture, University of Nebraska-Lincoln, Lincoln, NE 68583, USA; yavuzdelenn@gmail.com (Y.D.); semrapalali.sp@gmail.com (S.P.-D.); jinliang.yang@unl.edu (J.Y.); 2 Center for Plant Science Innovation, University of Nebraska-Lincoln, Lincoln, NE 68583, USA; gxu6@unl.edu; 3 Crop Science Department, Agricultural Institute of Slovenia, Hacquetova ulica 17, SI-1000 Ljubljana, Slovenia; mohamed.neji@kis.si

**Keywords:** sunflower, GWAS, flowering time, plant height, stem diameter, seed weight, kernel weight

## Abstract

The sunflower (*Helianthus annuus* L.) is one of the most essential oil crops in the world. Several component traits, including flowering time, plant height, stem diameter, seed weight, and kernel weight, determine sunflower seed and oil yield. Although the genetic mechanisms governing the variation of these yield-related traits have been studied using various approaches, genome-wide association studies (GWAS) have not been widely applied to sunflowers. In this study, a set of 342 sunflower accessions was evaluated in 2019 and 2020 using an incomplete randomized block design, and GWAS was conducted utilizing two complementary approaches: the mixed linear model (MLM) and the fixed and random model circulating probability unification (farmCPU) model by fitting 226,779 high-quality SNPs. As a result, GWAS identified a number of trait-associated SNPs. Those SNPs were located close to several genes that may serve as a basis for further molecular characterization and provide promising targets for sunflower yield improvement.

## 1. Introduction

Sunflower domestication likely began over 4000 years ago [1] in the interior mid-latitudes of eastern North America [2]. Various selection and breeding methods have contributed considerably to today’s sunflower varieties. Since the sunflower was domesticated, it has been used for multiple purposes in various industries. It is an essential oil crop that makes a substantial contribution to the world’s supply of edible oil. The sunflower is currently Europe’s second-most important oil crop, behind rapeseed, and the fourth-most important oil crop in the world [3], with its high-quality edible oil and dietary fiber being essential to a balanced human diet [4].

Because of the growing human population and other environmental concerns, the demand for sunflowers has been rising. To address this need, sunflower breeding and genetic studies have been conducted to improve sunflowers’ yield and quality parameters. Different techniques and approaches have been utilized to enhance agronomically important sunflower traits. However, the genetic basis for governing the yield traits remains largely unknown. Therefore, understanding the genetic mechanism of sunflower traits using GWAS has become one of the major ongoing objectives of many projects.

The sunflower is mostly grown for the seed (achene) production that is used in oil production. As a quantitatively inherited component of sunflowers, seed yield is highly influenced by environmental factors. It is dependent on the genetic potential of the cultivars as well as contributions from other yield-related factors like seed weight, plant height, head diameter, and so on [5,6,7,8,9,10]. Flowering time is also another factor affecting seed yield. It is mainly because flowering time has a big impact on local adaptation and reproduction—a fitness trait [11,12,13]. Sunflower populations exhibit significant variations in flowering time. Although a late flowering time was targeted in the early domestication of sunflowers [14], today’s modern sunflowers are flowering relatively early, which is intended for a high yield since early flowering offers resilience to drought stress [15,16]. Likewise, later flowering hybrids are heavily impacted by dry circumstances because of infrequent rain and scorching seasons [17]. Kaya et al. (2009) [18] indicated that hybrids that were earlier in flowering and had a longer physiological maturity length than 107 days promoted seed yield in sunflowers. They also highlighted the fact that plant height and seed volumes are critical parameters for oil-type sunflower hybrids to perform better in seed and oil production. Sunflower populations exhibit a wide range of variation in terms of plant height, with the short (~50 cm) and long (above 3 m) varieties. Many studies have reported that reduced plant height has many benefits, including increased plant density, resistance to lodging and certain diseases, etc. Still, it positively affects yield and is recognized as a precious yield trait [5,6,7,19,20,21]. Therefore, plant height is one of the selection criteria for breeding purposes. The enhanced combining ability of the hybrid parents and selection for adaptability to particular conditions, such as durable plant stem, is linked to the critical advancements in enhancing grain production in sunflowers, according to Fernandez et al. (2009) [22]. As another important yield parameter, the stem diameter was reported to be related to seed yield in sunflowers. Shankar et al. (2006) [23] and Habib et al. (2007) [24] reported positive correlations of stem diameter with sunflower seed yield per plant. Similarly, kernel weight is a well-known trait directly correlated with sunflower seed and oil yield and has a positive effect on oil yield through seed yield [25].

In this study, we collected five agronomically important sunflower traits, namely flowering time, plant height, stem diameter, seed weight, and kernel weight, from yield trials in 2019 and 2020. After combining the genotypic data we previously generated using the tunable genotyping-by-sequencing (tGBS) method, GWAS was performed by utilizing MLM and farmCPU methods to associate the SNP markers with traits. As a result, trait-associated SNPs and candidate genes were identified, which provide valuable targets for further sunflower improvement.

## 2. Materials and Methods

### 2.1. Plant Materials

In this study, a set of 342 sunflower accessions were evaluated for essential yield components influencing oil and seed production, which were flowering time, plant height, stem diameter, seed weight, and kernel weight. The accessions were obtained from the United States Department of Agriculture, Agricultural Research Center (USDA-ARS), North Central Regional Introduction Station (NCRPIS) in Ames, Iowa, USA. The accessions have been assembled from diverse locations worldwide, tested, and maintained to be distributed to researchers or institutions in this gene bank. These 342 sunflower accessions were selected randomly based on different geographical locations on six continents to create diversity in the population. Based on the information and GPS coordinates of the collection associated with individual accessions in the USDA’s Germplasm Resources Information Network (GRIN) “https://www.ars-grin.gov (accessed on 9 October 2021)”, most of the genotyped sunflower accessions used in this study originated from North America, Europe, and Asia, with a small number of accessions from South America, Africa, and Australia.

### 2.2. Field Experiment and Phenotyping

The accessions were planted at the Agronomy and Horticulture Research Station in Havelock, University of Nebraska-Lincoln, in 2019 (40°51′15.9″ N, 96°36′42.6″ W) and 2020 (40°51′26.5″ N, 96°36′53.4″ W) using an incomplete randomized block design. The field experiment had two main blocks, each with four split plots and three checks (PI 432513) per split-plot. Each genotype was planted in a 3.6 m long single row with 0.75 m row spacing and an alleyway of 0.9 m. A density of about 45,000 plants per hectare was achieved by planting twelve seeds per row. Phenotypic data collection for the flowering time began with the flowering of the first accession, and the field was visited every morning throughout the flowering period of the accessions. Flowering time was determined when 50% of plants in an accession line bloomed. After the plants’ flowering, phenotypic data for plant height and stem diameter were collected. Once the plants matured, the seeds were harvested, and the bulked seeds were dried, counted, and weighed to measure 100 seed weights (in grams). After removing the hull, 100 kernel weights (in grams) were also noted for further analysis. The phenotype data was collected from three representative plants per plot, excluding edge plants.

### 2.3. Phenotypic Data Processing and Heritability Calculation

Best Linear Unbiased Prediction (BLUP) was performed for all traits by using the lme4 package [26] in R (v 4.2.0) [27] to predict the additive genetic value of each genotype in the population to be used in GWAS. In the BLUP calculation, genotype, block, split-plot, year, and genotype-by-year interaction were considered as random effects by fitting the following model, using phenotype data from 2019 and 2020: Y~(1|genotype) + (1|block) + (1|split-plot) + (1|year) + (1|genotype:year) + error, where Y represents the phenotype (flowering time, plant height, stem diameter, seed weight, and kernel weight). In the model,

*yijkrl* = *µ* + *qi* + *tl* + *qi* ∗ *tl* + *bjrl* + *sjkrl* + *qrl* + *ε*

where *yijkrl* refers to the phenotypic value of the *ith* genotype evaluated in the *kth* split-plot of the *jth* block of *rth* replicate nested within the lth year; *µ* is the overall mean; *qi* is the random effect of the *ith* genotype; *tl* is the random effect of the *lth* year; *qi*∗*tl* is the random effect of the *ith* genotype with the *lth* year interaction; *bjrl* is the random effect of the *jth* block of the *rth* replicate within the *lth* year; *sjkrl* is the random effect of the *kth* split-plot of the *jth* block of the *rth* replicate within the *lth* year; *qrl* is the random effect of the *rth* replicates nested within the *lth* year; *ε* is the random residual error.

Broad-sense heritability (*H*^2^) was calculated based on the equation *H*^2^ = *V_G_*/*V_P_* [28,29], where *V_G_* is total genetic variance, *V_P_* is total phenotypic variance (*V_P_* = *V_G_* + *V_E_*), and *V_E_* is phenotypic variance due to environmental factors. Regarding this, the broad-sense heritability of interested traits was calculated for the combined environments of 2019 and 2020 using the following equation:

$$H^{2}=\frac{\sigma_{g}^{2}}{\sigma_{g}^{2}+\frac{\sigma_{gxy}^{2}}{n}+\frac{\sigma_{e}^{2}}{nr}}$$

where *σ*^2^*_g_* is the components of variance for genotype, *σ*^2^*_e_* is the components of variance for the environment, *σ*^2^*_gxy_* is the components of variance for genotype by year interaction, *n* is the number of years, and *r* is the number of replications.

### 2.4. Collection of Leaf Samples and DNA Extraction for Genotyping

Sunflower accessions were planted and grown in a greenhouse to collect leaf samples for DNA extraction. The accessions were grown in 10 × 10 cm pots, which were filled with a standard greenhouse mix consisting of 5 gallons of peat, 3 gallons of soil, 2.5 gallons of sand, and 2.5 gallons of vermiculite. After a growth period of two weeks, two samples of young leaves were taken at the V4 stage, each weighing 600–700 mg. The samples were then stored in sterile Eppendorf tubes at a temperature of −80 °C to prevent damage to the leaves and to obtain high-quality DNA. To extract the DNA, the leaf samples were first freeze-dried using a lyophilization machine. The dried samples were then sent to Freedom Markers “https://www.freedommarkers.com/” (accessed on 3 February 2021) for DNA extraction and genotyping. For DNA extraction, the BioSprint MagAttract 96 DNA Plant Core Kit from QIAGEN was used and then quality-checked using a PicoGreen kit on an Eppendorf Plate Reader. The accession “PI 490282” was added once to each healthy plate as a control to ensure genotyping quality.

### 2.5. SNP Genotyping

The genotyping of 285 sunflower accessions was performed using tGBS genotyping by sequencing technology. Initially, quality control measures were performed, followed by the pooling of samples and the generation of tGBS libraries. Subsequently, the raw sequencing data was de-barcoded, and low-quality base pairs at the beginning and end of each read were eliminated by quality trimming. The resulting reads were aligned to the Helianthus Annuus HanXRQr2.0-SUNRISE reference genome [11] to identify genotypes and polymorphic markers. Due to a high missing rate, 11 of the 285 initially selected samples were excluded from further analysis. Genotyping was performed by Freedom Markers using the restriction enzyme Bsp1286I [30], and an Illumina HiSeq X instrument with 2 × 150 bp paired-end reads, resulting in two sets of SNP sites. The first set comprised 503,188 SNPs (ALL SNPs), while the second set was more stringent and of higher quality, with 247,008 SNP sites, of which each SNP site was genotyped in at least 50% of the samples (MCR50 SNPs), with an average of 22 tGBS reads per SNP/genotyped sample. These criteria were adhered to while filtering ALL SNPs: (1) there must be a minimum calling rate of 20%; (2) the allele number must equal 2; (3) there should be at least two genotypes; (4) the frequency of the minor allele must be at least 5%; (5) the range of the heterozygosity rate is 0% (2 × Frequencyallele1 × Frequencyallele2 + 20%). The filtering criteria for the MCR50 SNPs were also the same as the filtering criteria for ALL SNPs except for the minimum calling rate, which should be more than 50%. Imputation was performed for the MCR50 SNPs within each genotype to close data gaps.

### 2.6. Processing of Sequence Data

The fastq sequence data were trimmed for quality based on PHRED quality scores [31,32], removing regions of low quality. The trimming parameters adopted from the “LUCY2 v2.20” software [33,34] included scanning the reads and removing nucleotides with quality scores below 15. In addition, all windows with an average PHRED quality score below 15 were trimmed. Reads were aligned to the HanXRQr2.0-SUNRISE reference genome of sunflowers using GSNAP [35]. Only readings aligning to a single site in the reference genome were used for SNP calling. For the subsequent analyses, only securely and unambiguously mapped reads that met specific criteria regarding mismatches and base tails were used. Then, SAMtools v1.16 [36] was used to convert the formats of the alignment files. Polymorphic sites that differed from the reference genome were identified from reads that matched the sunflower reference genome. SNP markers were genotyped using criteria for homozygous and heterozygous SNPs, considering read support and quality scores.

#### Detecting Polymorphic Sites

SNPs were found using the coordinates of unique and confident alignments that met the filtering requirements. Following a thorough examination of the polymorphisms at each putative SNP location, putative homozygous and heterozygous SNPs were independently identified in each sample. For this purpose, the criteria for homozygous SNPs were used following those points: (1) at least 80% of all aligned reads covering that site must support the most prevalent allele; (2) to support the most frequent allele, at least five distinct readings are required; (3) the initial and final three base pairs of every read were stripped of polymorphisms; (4) the minimum PHRED base quality value for each polymorphic base is 20, which corresponds to a 61% error rate. As for the criteria for heterozygous SNPs, it covered the following: (1) at least 30% of all aligned reads covering that site must support each of the two most prevalent alleles; (2) for each of the two most prevalent alleles, there must be a minimum of five distinct readings; (3) at least 80% of all aligned reads covering that nucleotide location must be accounted for by the sum of reads from the two most prevalent alleles; (4) each quality-trimmed read’s initial and last 3 bp of polymorphisms were disregarded; (5) a minimum PHRED base quality rating of 20 (≤1% error rate) is required for every polymorphic base. A single nucleotide was considered homozygous at an SNP site in a diploid sample if at least five reads supported the predominant common allele at that site and at least 90% of all aligned reads covering that site shared the same nucleotide. Similarly, in a given diploid sample, a single nucleotide polymorphism (SNP) was deemed heterozygous if at least two reads supported a minimum of two distinct alleles, if the sum of the two allele types’ alignment for that site exceeded 20%, and if the total number of reads supporting those two alleles equals or exceeds five and when those readings account for at least 90% of all reads that cover the site.

### 2.7. SNP Analysis

PCA was conducted using TASSEL v5 [37] to test the genetic variations in the dataset. The scree plot was generated using the eigenvalues of 10 principal components to visualize the proportion of variance explained by each PC. PLINK 1.9 [38] software, integrated with R [36], was used to estimate the linkage disequilibrium (LD) with *r*^2^ statistics [39], build LD decay, and perform the minor allele frequency (MAF). Additionally, TASSEL v5 was used to estimate the individual kinship in the dataset.

### 2.8. Genome-Wide Association Analysis

In this study, 247,008 SNPs were initially detected and filtered down to 246,671 SNPs by removing excessive alignments on unplaced genomic scaffolds. For marker–trait association analysis, the SNP set was further filtered by removing SNPs with an MAF ≤ 0.05 across 274 individuals, resulting in a set of 226,779 SNPs. This marker set was used for GWAS analysis with two models: MLM (Q + K) [40] and farmCPU [41]. Both models were run using the R package “rMVP” (v1.0.6) [42]. In the MLM model, the kinship matrix [43] and the first three principal components were fit as covariates to control for the confounding effects of population structure. The significant association threshold was 2.2 × 10^−7^ (0.05/n, n = 226,779) following a previous study [44]. For the farmCPU model, the kinship matrix calculated internally by the algorithm was fitted as random effects in addition to the first three PCs as covariates. GWAS results were plotted in rMVP using Manhattan plots and Q-Q plots.

## 3. Results

### 3.1. Correlation, Heritability, and BLUP Value Calculation

**Figure 1:**
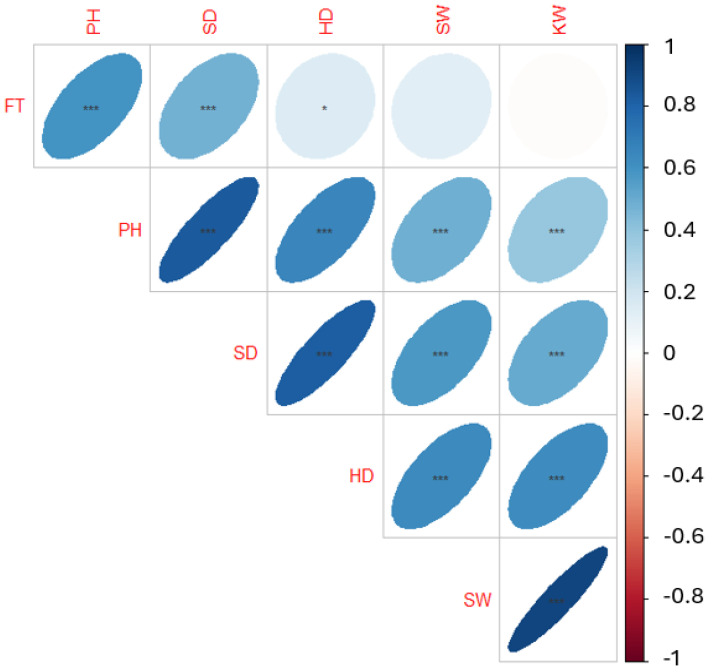
Correlation plot of yield component traits. The six traits are flowering time (FT), plant height (PH), stem diameter (SD), head diameter (HD), seed weight (SW), and kernel weight (KW). Asterisk indicates statistical significance level, with *** < 0.001, and * < 0.05.

We obtained 342 geographically dispersed sunflower accessions from the North Central Regional Introduction Station (NCRIS) of the United States Department of Agriculture. Using an incomplete block design, these accessions were planted on the Havelock Research Farm at the University of Nebraska-Lincoln in 2019 and 2020. The phenotypic data for various traits were manually collected from three representative plants per plot. A correlation analysis (Pearson’s correlation) was conducted to determine the relationships between these traits. Results suggested strong correlations between some characteristics (Figure 1 and Table A1). It was observed that while the seed and kernel weights were not correlated with flowering time, the head diameter assessed in another study [45] was found to be significantly associated with a *p* < 0.05. All other correlations between traits were statistically significant (*p* < 0.001).

Estimates of broad-sense heritability (*H*^2^), an essential parameter in the breeding process, give information on the ratio of genetic and additive effects on phenotypic variance. After analyzing the data for two years, we estimated the broad-sense heritability of the five traits, and the estimated *H*^2^ values were 0.94, 0.89, 0.88, 0.63, and 0.61 for flowering time, plant height, stem diameter, seed weight, and kernel weight, respectively. Further, we combined the data from two years to calculate the Best Linear Unbiased Predictions (BLUP) values for these traits. The BLUP values for flowering time, plant height, stem diameter, seed weight, and kernel weight ranged from 55.7 to 91.7 with a mean of 66.7, from 88.8 to 281.7 with a mean of 167.1, from 10.9 to 31.7 with a mean of 20.6, from 1.25 to 14.81 with a mean of 7.06, and from 1.02 to 8.83 with a mean of 4.87, respectively.

### 3.2. SNP Genotyping and Population Structure Analysis

**Figure 2:**
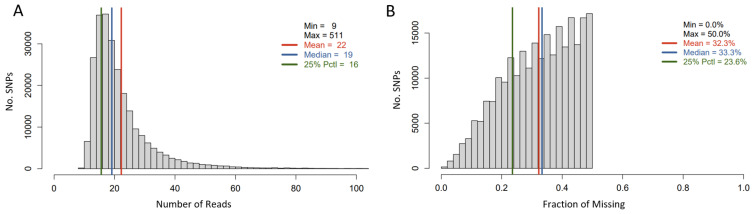
Histogram of Read number per SNP site per genotyped sample (**A**) and that of average missing data rate per SNP Site (**B**).

The tGBS approach was used for genotyping a subset of 285 out of 342 sunflower accessions. After SNP calling, the SNPs with a minimum calling rate of less than 50% were removed, and 11 out of 285 accessions were eliminated due to a high individual missing rate (i.e., >90%). This resulted in 247,008 SNPs being retained for 274 sunflower accessions. The average number of reads per SNP site per sample was 22, and the average missing rate for this SNP set was 32.3% (Figure 2).

**Figure 3:**
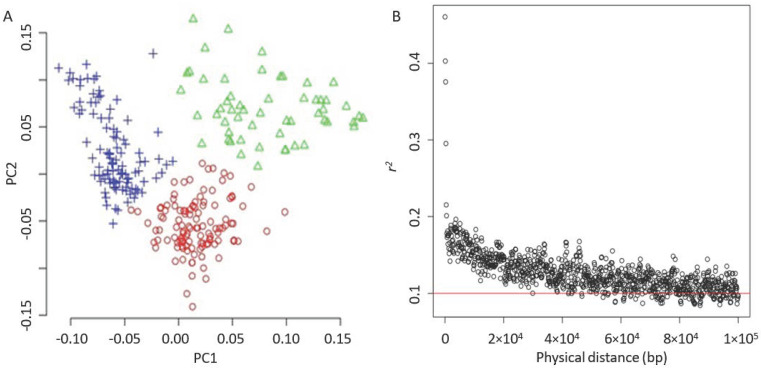
Principle component analysis and LD decay. (**A**) PCA plot. The accessions were clustered by using the simple k-means algorithm with the three groups. (**B**) LD decay. The red solid line indicates a threshold of 0.1.

**Figure A1:**
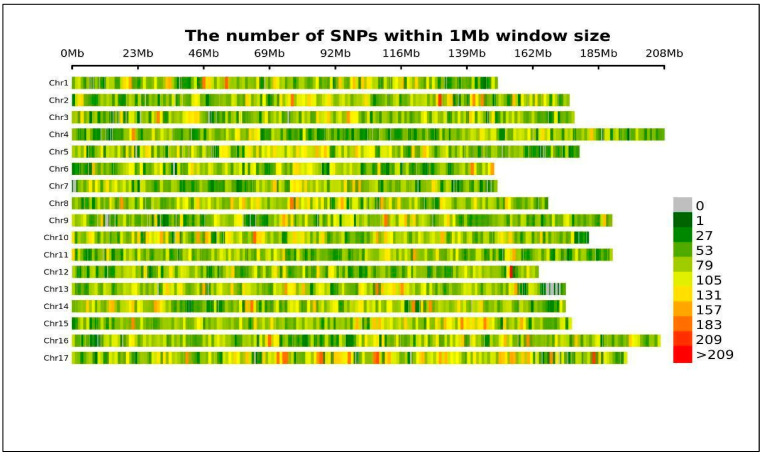
Distribution of SNPs among the 17 chromosomes of sunflowers.

After removing unnecessary alignments on 147 unplaced genomic scaffolds, the SNP set was filtered to collect 246,671 SNPs. An MAF of less than 0.05 was filtered, resulting in 226,779 SNPs. These SNPs were evenly distributed among the 17 chromosomes of sunflowers (as shown in Figure A1). The k-means algorithm was used to identify three groups, indicating that our sunflower accessions probably comprise three subpopulations (Figure 3A). The principal component analysis showed that the first principal component (PC) accounted for approximately 5% of the variance, and the top 10 PCs explained 25% overall.

Using the filtered SNP set, it was found that the linkage disequilibrium (LD) decayed quickly in the population, with LD decreasing from *r*^2^ = 0.2 to 0.15 as the average pairwise SNP distance increased from 18 kb to 30 kb. When the LD was *r*^2^ = 0.1, the average physical distance between two SNPs was approximately 220 kb (Figure 3B), which aligns with the previous research [46]. The principal component analysis indicated that the first principal component (PC) accounted for approximately 5% of the variance, and the top 10 PCs explained 25% overall. 

### 3.3. Genome-Wide Association Study

The GWAS analysis was conducted using a set of 226,779 SNPs with two different statistical methods, MLM and farmCPU. The quantile-quantile (Q-Q) plots suggested that the population structure was well controlled for GWAS analyses in both models. As a result, significant trait-associated SNPs were identified and represented in red by setting the threshold as −log_10_(*p*) = 5.

#### 3.3.1. Flowering Time

**Figure 4:**
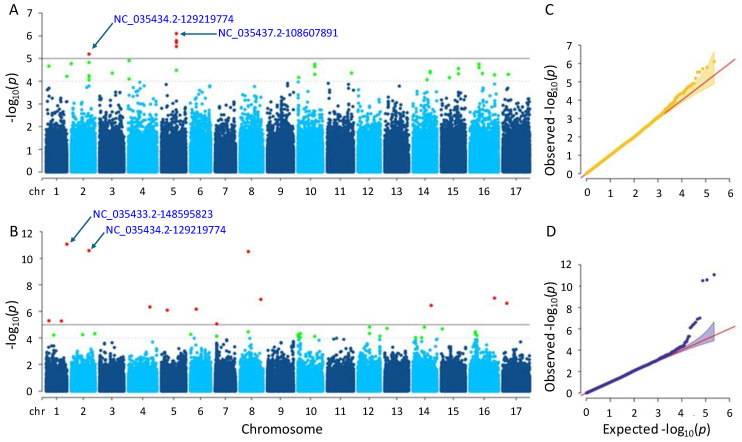
Manhattan plots and Q-Q plots for flowering time. (**A**) Manhattan plot for MLM. (**B**) Manhattan plot for farmCPU. (**C**) Q-Q plot for MLM. (**D**) Q-Q plot for farmCPU. The vertical and horizontal axes in the Manhattan plots indicate the *p*-values on the −log10 scale and the chromosome numbers, respectively. The points represent the SNPs; the horizontal lines are the significance thresholds. The Q-Q plots on the right side of the Manhattan plots illustrate the deviation of the *p*-values from the null hypothesis; the vertical and horizontal axes are observed and expected *p*-values, respectively, with the red lines representing the null hypothesis.

A total of six and thirteen significant SNPs were detected for the flowering time trait using the MLM and farmCPU methods, respectively (Tables S1 and S2). In the MLM method, the SNPs were found on chromosomes 2 and 5, while in the farmCPU method, they were found on ten different chromosomes (Figure 4). Notably, a significant SNP accumulation on chromosome 5 was identified in the MLM method, with *p*-values ranging from 7.89 × 10^−7^ to 6.40 × 10^−6^. The *p*-values of the significant SNPs detected by the farmCPU method also ranged from 8.60 × 10^−12^ to 8.81 × 10^−6^. The most significant SNPs identified by MLM and farmCPU were NC_035437.2-108607891 on chromosome 5 and NC_035433.2-148595823 on chromosome 1, respectively.

After examining gene annotation information, four and thirty-eight genes were identified as closely located to the significant SNPs identified by the MLM and farmCPU methods, respectively (See Tables S3 and S4 for more details). Herein, a significant SNP ‘NC_035434.2-129219774’ on chromosome 2 was detected by both models, MLM and farmCPU. According to the MLM method, this SNP was closely located in the two uncharacterized genes, which are LOC118486721 and LOC110920267. In addition, some genes detected by trait-associated SNPs using the MLM method were functionally annotated as follows: membrane-associated kinase regulator 2, regulating root gravitropic bending and TMK-dependent rapid auxin signaling (LOC110920267); CADH6, biosynthesizing lignin (LOC110941024); and extensin-like, playing a role on physical characteristics of the plant cell wall (LOC110943450). The others detected by farmCPU were as follows: aquaporin PIP2-7, leading the transport of water across membranes, as well as signaling and stress reactions (LOC110925968); O-fucosyltransferase 28, glycosylating numerous substrates and regulating protein–protein interactions involved in cell adhesion and cell–cell communication (LOC110865320); L-type lectin-domain containing receptor kinase VIII.1, participating in a range of developmental processes and plant defense reactions (LOC110944662); auxin-repressed 12.5 kDa protein, responsive to auxins—a hormone that controls plant growth and development—as well as hormones involved in defense response to biotic stress (LOC110935339); and DI193, playing significant role in abiotic stress (LOC110944327).

#### 3.3.2. Plant Height

**Figure 5:**
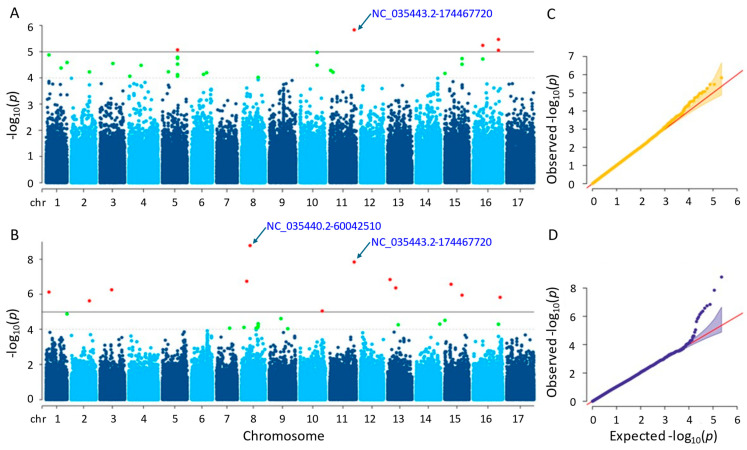
(**A**) Manhattan plot for MLM. (**B**) Manhattan plot for farmCPU. (**C**) Q-Q plot for MLM. (**D**) Q-Q plot for farmCPU. The vertical and horizontal axes in the Manhattan plots indicate the *p*-values on the −log10 scale and the chromosome numbers, respectively. The points represent the SNPs; the horizontal lines are the significance thresholds. The Q-Q plots placed on the right side of the Manhattan plots illustrate the deviation of the *p*-values from the null hypothesis; the vertical and horizontal axes are observed and expected *p*-values, respectively, with the red lines representing the null hypothesis.

It was found that after conducting GWAS, six and twelve significant single-nucleotide polymorphisms (SNPs) were identified by the MLM and farmCPU methods, respectively (Tables S1 and S2). The considerable SNPs of the MLM method were observed on chromosomes 5, 11, and 16, whereas those of the farmCPU method were placed on nine different chromosomes (Figure 5). The *p*-values of the significant SNPs ranged from 1.48 × 10^−6^ to 8.83 × 10^−6^ in the MLM method and from 1.65 × 10^−9^ to 9.05 × 10^−6^ in the farmCPU method. The MLM and the farmCPU methods found the most significant SNPs, which were NC_035443.2-174467720 and NC_035440.2-60042510, respectively. Additionally, 12 and 40 genes were detected for the plant height that were placed close to the significant SNPs identified by the MLM and farmCPU methods, respectively (Tables S3 and S4). Based on the GWAS results of both methods, a considerable SNP, NC_035443.2-174467720, on chromosome 11 was identified as being related to the plant height of the sunflower. This SNP was closely located to three genes, namely LOC110888769, LOC110888770, and LOC118484155, regarding the farmCPU method. When we evaluated the MLM method, the genes ‘LOC118484414’, ‘LOC118484415’, and ‘LOC110888776’ were closely located to this SNP.

Many of the genes detected by both models were annotated as coding proteins. Some of those proteins encoded by the genes detected by the MLM method were as follows: BAHD acyltransferase, BIA1, which acylates primary and specific secondary metabolites in plants (LOC110888776); E3 ubiquitin-protein ligase AIRP2, acting in abscisic acid (ABA) and dehydration stress (LOC110915864); NAC domain-containing protein 75-like, controlling abiotic and biotic stress reactions in plants (LOC110916982); heterogeneous nuclear ribonucleoprotein Q, which engages in the response of the plant immune system (LOC110918080); CADH6, participating in the biosynthesis of lignin (LOC110941024); extensin-like (LOC110943450), having an impact on the physical properties of the plant cell wall. Some other proteins of the genes identified by farmCPU method were as follows: cold-responsive protein kinase 1, effecting the plant’s response to cold stress (LOC110883118); villin-5, contributing significantly to the control of actin dynamics in eukaryotic cells as well as the assembly of higher-order structures from actin filaments (LOC110886186); peroxidase 16 functioning elimination of H2O2, oxidation of harmful reductants, lignin production and degradation, suberization, auxin catabolism, and reaction to environmental stressors (LOC110886188); NOP53 playing role on ribosome biogenesis and nuclear stress (LOC110886190); NAC domain-containing protein 7, promoting the expression of genes linked to secondary wall biosynthesis, xylem development, and transcription factors related with the secondary walls (LOC110897930); extensin-like, acting on physical characteristics of the plant cell wall (LOC110900877); F-box/FBD/LRR-repeat protein At1g13570-like with various functions including growth and development of plants, in addition to physiological and biochemical responses (LOC110920226, LOC110920484, LOC110920485, LOC110920486, LOC110920487, LOC110920488, LOC110920489, LOC110920490, LOC110920491, LOC110920492, LOC110920493, and LOC110920494); importin-4, controlling gene delivery by improving nuclear retention and polyplex chromatin deposition (LOC110929478); DRM1 that is responsive to auxins, a hormone that controls plant growth and development, as well as hormones involved in defense response to biotic stress (LOC110935339); DI193, playing significant role in abiotic stress (LOC110944327).

#### 3.3.3. Stem Diameter

**Figure 6:**
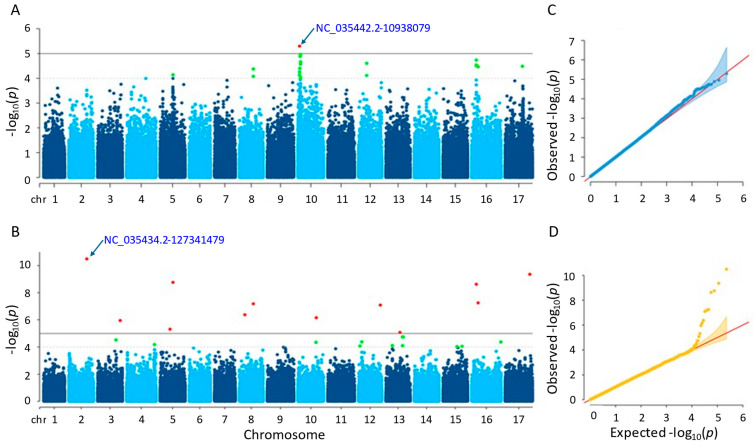
(**A**) Manhattan plot for MLM. (**B**) Manhattan plot for farmCPU. (**C**) Q-Q plot for MLM. (**D**) Q-Q plot for farmCPU. The vertical and horizontal axes in the Manhattan plots indicate the *p*-values on the −log10 scale and the chromosome numbers, respectively. The points represent the SNPs; the horizontal lines are the significance thresholds. The Q-Q plots on the right side of the Manhattan plots illustrate the deviation of the *p*-values from the null hypothesis; the vertical and horizontal axes are observed and expected *p*-values, respectively, with the red lines representing the null hypothesis.

The results of the GWAS analysis revealed that the MLM method detected one significant SNP, while the farmCPU method detected twelve significant SNPs (Tables S1 and S2). The SNP detected (NC_035442.2-10938079) by the MLM method was located on chromosome 10, with a *p*-value of 5.07 × 10^−6^. On the other hand, the significant SNPs detected by the farmCPU method were located on nine different chromosomes, with *p*-values ranging from 3.18 × 10^−11^ to 8.48 × 10^−6^. The most significant SNP identified by farmCPU was NC_035434.2-127341479 on chromosome 2 (Figure 6). Regarding the GWAS results, the significant SNPs detected by both methods were not shared.

Following gene annotation, it was discovered that four genes situated close to the essential SNPs uncovered by the MLM technique were linked to the sunflower stem diameter trait. Comparably, it was found that 36 genes related to the same characteristic were situated close to the critical SNPs that the farmCPU technique had identified. For further information, please see Tables S3 and S4.

The characterized genes detected by the MLM and farmCPU methods functioned in coding essential proteins. Those proteins are as follows: NPGR2, calmodulin-binding protein, acting in pollen germination (LOC110883981); endoglucanase 6, enabling hydrolase activity and carbohydrate binding (LOC110921587); bidirectional sugar transporter SWEET4, acting as sugar transporter in plant development (LOC110930262, LOC110928153); cytochrome c oxidase subunit 5C-2, which is a terminal oxidase involved in electron transport within the mitochondria (LOC110930263); transcription factor bHLH68, contributing to the regulation of ABA homeostasis and drought stress tolerance (LOC110940444); copper transport protein ATX1-like, acting in maintaining copper homeostasis by providing resistance to excess copper and subclinical copper deficit during the vegetative stage (LOC110870432); GDSL esterase/lipase CPRD49, participating in the control of morphogenesis, defense response, synthesis of secondary metabolites, and plant development (LOC110872698); UDP-glycosyltransferase 84B1, which has minimal in vitro activity with quercetin 7-O-glucosyltransferase, responsible for the plant’s IAA-glc formation, and plays role in auxin homeostasis and plant development (LOC110885124); and proline-rich protein 36-like, functioning in developmental stages and stress responses (LOC110919168).

#### 3.3.4. Seed Weight

**Figure 7:**
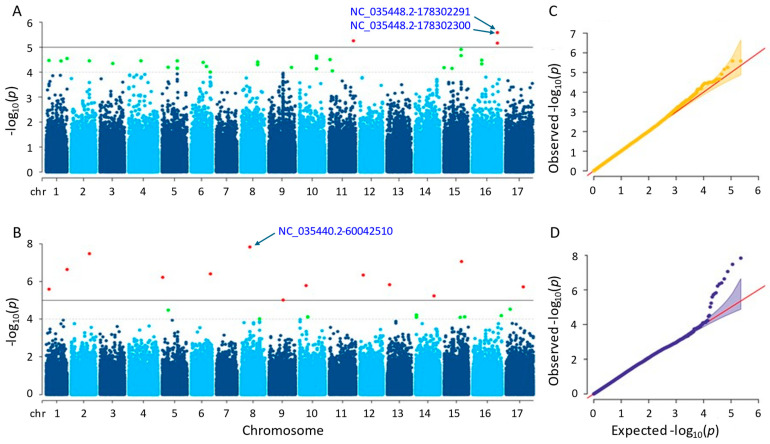
(**A**) Manhattan plot for MLM. (**B**) Manhattan plot for farmCPU. (**C**) Q-Q plot for MLM. (**D**) Q-Q plot for farmCPU. The vertical and horizontal axes in the Manhattan plots indicate the *p*-values on the −log10 scale and the chromosome numbers, respectively. The points represent the SNPs; the horizontal lines are the significance thresholds. The Q-Q plots on the right side of the Manhattan plots illustrate the deviation of the *p*-values from the null hypothesis; the vertical and horizontal axes are observed and expected *p*-values, respectively, with the red lines representing the null hypothesis.

Significant SNPs were identified using the MLM and farmCPU methods. The MLM method detected four significant SNPs on chromosomes 11 and 16, with *p*-values ranging from 2.62 × 10^−6^ to 6.90 × 10^−6^ (Figure 7A). The farmCPU method identified 13 significant SNPs on 12 different chromosomes, with *p*-values ranging from 1.45 × 10^−8^ to 9.77 × 10^−6^ (Figure 7B). Tables S1 and S2 provide additional details on the SNPs identified by both methods. By utilizing the MLM method, the two most significant SNPs (NC_035448.2-178302291 and NC_035448.2-178302300) with the exact *p*-values were detected on chromosome 16. The most significant SNP in the farmCPU method was NC_035440.2-60042510 on chromosome 8. As a result of the GWAS, both MLM and farmCPU methods identified significant SNPs associated with seed weight; however, no significant SNPs were shared. After gene annotation, eight and thirty-eight genes associated with sunflower seed weight were located close to the significant SNPs detected by the MLM and farmCPU methods, respectively (Tables S3 and S4). 

The proteins coded by the characterized genes that we detected in this study by MLM and farmCPU methods were as follows: BAHD acyltransferase BIA1, involving in plant growth (LOC110888776); NAC domain-containing protein 75-like, controlling abiotic and biotic stress reactions (LOC110916982); heterogeneous nuclear ribonucleoprotein Q, engaging in the response of the plant immune system (LOC110918080); ethylene-responsive transcription factor WIN1, promoting the development of cuticles by expressing the enzymes necessary for the creation of wax providing tolerance to drought (LOC110884445); auxin-repressed 12.5 kDa protein, responsive to auxins controlling plant growth and development (LOC110935339); protein dehydration-induced 19 homolog 4, enhancing drought tolerance (LOC110944327); probable membrane-associated kinase regulator 2, acting in the regulation of plant development (LOC110920267); and probable 2-oxoglutarate-dependent dioxygenase At5g05600, initiating the first stage of the flavonoid biosynthesis pathway that produces anthocyanins and flavanols (LOC110885674), respectively.

#### 3.3.5. Kernel Weight

**Figure 8:**
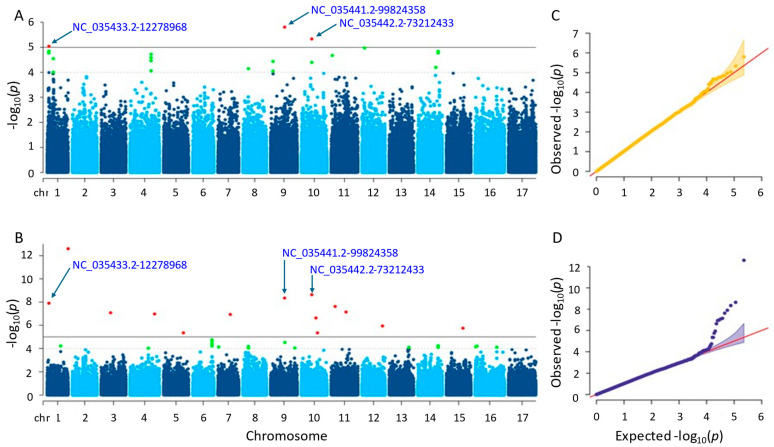
(**A**) Manhattan plot for MLM. (**B**) Manhattan plot for farmCPU. (**C**) Q-Q plot for MLM. (**D**) Q-Q plot for farmCPU. The vertical and horizontal axes in the Manhattan plots indicate the *p*-values on the −log10 scale and the chromosome numbers, respectively. The points represent the SNPs; the horizontal lines are the significance thresholds. As for the Q-Q plots placed on the right side of the Manhattan plots, they illustrate the deviation of the *p*-values from the null hypothesis; the vertical and horizontal axes are observed and expected *p*-values, respectively, with the red lines representing the null hypothesis.

Following GWAS, three and fourteen significant SNPs were found using the two separate approaches, farmCPU and MLM (Figure 8; Tables S1 and S2). The SNPs found on chromosomes 1, 9, and 10 were detected using the MLM approach with *p*-values ranging from 1.57 × 10^−6^ to 9.12 × 10^−6^. Conversely, the SNPs identified by the farmCPU technique were found on ten distinct chromosomes, with *p*-values ranging from 2.53 × 10^−13^ to 4.49 × 10^−6^. Following gene annotation, it was discovered that, concerning the critical SNPs reported by the MLM and farmCPU approaches, four and forty-six genes were linked to sunflower kernel weight (see Tables S3 and S4). When we compared the results, we found that three significant SNPs, NC_035433.2-12278968, NC_035441.2-99824358, and NC_035442.2-73212433, on chromosomes 1, 9, and 10 were shared by both models. The SNP ‘NC_035433.2-12278968’ was closely located to two genes (LOC110863868 and LOC110863881) in the results of both methods. Similarly, another SNP, NC_035441.2-99824358, was closely located to two genes, LOC110875903 and LOC110875902.

The genes that were identified by the MLM method functioned in protein coding. Those proteins were the cationic amino acid transporter 1 (LOC110863868) and 30S ribosomal protein S13, chloroplastic (LOC110863881). The others detected by farmCPU were as follows: ethylene-responsive transcription factor WIN1, promoting the development of cuticles by expressing the enzymes necessary for the creation of wax that contributes abiotic stress response (LOC110884445); protein JINGUBANG, which is a negative pollen germination regulator playing a role in proper tube growth (LOC110941472); protein far-red elongated hypocotyl 3-like, promoting floral meristem determinacy, regulates chlorophyll biosynthesis, increases leaf longevity (LOC110918945); serine/threonine-protein phosphatase 7 long form homolog, functioning in the control of light, stress, hormone signaling, and the metabolism, cell cycle, and development (LOC110918946); thermospermine synthase ACAULIS5, supporting accurate xylem specification by controlling the xylem elements’ lifespan and keeps the components of the xylem vessel from premature death (LOC110885237); glyceraldehyde-3-phosphate dehydrogenase, cytosolic and has a significant impact on the cellular synthesis of energy, carbohydrate metabolites, and reductants (LOC110885235); putative nuclease HARBI1, a transposase-derived protein that may have nuclease activity (LOC110881343); phosphatidylinositol 4-phosphate 5-kinase 8, controlling a variety of cellular functions (LOC110884744); phosphatidylinositol/phosphatidylcholine transfer protein SFH9, facilitating the movement of phosphatidylcholine and phosphatidylinositol across membranes in vitro (LOC110915404); transmembrane protein 258-like, which engages in a variety of physiological processes in plants, such as energy conversion, substance transport, and signal transduction (LOC110893221); BRCA1-associated RING domain protein 1-like, functioning in DNA repair (LOC110892343); and ubiquitin carboxyl-terminal hydrolase 12, which is a positive regulator of root meristem growth that, in conjunction with UBP13, inhibits the RGF1 hormone peptide-induced ubiquitination and turnover of RGFR1 (LOC110893946).

## 4. Discussion

Detecting genetic variations that are statistically linked to a specific trait is accomplished through the use of the GWAS research methodology. The two standard techniques for GWAS are MLM and farmCPU. They employ a mixed model framework with different computational approaches, population structure management tactics, false-positive rate balancing procedures, and power strategies. MLM is the most often used model for association research, taking into consideration population structure and family relatedness [40,47]. Adjusting association tests to control false positives involves compromising true positives, which is why population structure and family relatedness are included in MLM models [41]. However, this model can potentially provide false-negative results because it overfits to the point where potentially significant connections are overlooked [48]. Another method, farmCPU, was created to consider both familial relatedness and population structure in GWAS. Fixed and random influences are integrated to overcome the confounding effects of population structure and familial relatedness. It resolves false positives efficiently while maintaining the integrity of true positives [41]. Based on the assumption of an even distribution of quantitative trait nucleotides, FarmCPU divides MLM into a computationally expensive REM (random effect model) and a computationally efficient FEM (fixed effect model) [41]. Both models have some advantages and disadvantages. FarmCPU is appropriate for investigations involving many markers or genotypes because it manages large-scale genetic information quickly. However, compared to MLM, it might not be as flexible because it is meant for particular dataset types and might not be appropriate for all genetic analyses. Similarly, MLM can handle various genetic data formats, such as markers with varying allele frequencies and quantitative and categorical features. However, significant computational power may be needed while dealing with big datasets or complicated models. Some studies indicate that the farmCPU method performed better than the MLM method [48,49,50], though MLM was also used successfully in many genome-wide association studies for various plant species [51,52,53]. In summary, choosing the method for GWAS analysis depends on the dataset’s specifics and the research’s goals.

The Q-Q plot analysis is a widely used method for assessing whether models control for false positives and false negatives, which displays the observed negative-log association probability values (Y-axis) against the expected negative-log association probability (X-axis) for all markers [49,54,55,56]. When we evaluated the two models used in this study, the farmCPU model performed better than the MLM model in controlling false positives and false negatives, exhibiting sharp upward deviated tails for all GWAS results of the studied traits (Figure 4, Figure 5, Figure 6, Figure 7 and Figure 8C,D).

Our study contributes significantly to the ongoing efforts to unravel the genetic determinants of key agronomic traits in sunflowers. Through a comprehensive analysis encompassing GWAS, we aimed to deepen our understanding of the genetic architecture underlying flowering time, plant height, stem diameter, seed weight, and kernel weight. Our findings reinforce and extend previous research, shedding light on the intricate interplay between these traits and their collective impact on sunflower yield.

Herein, many significant SNPs associated with the studied traits were identified. We detected the same significant SNPs related to seed weight, plant height, and flowering time in the farmCPU method, which were NC_035433.2-19748244, NC_035434.2-129219774, and NC_035440.2-60042510. The significant SNP, NC_035434.2-129219774, was also detected by the MLM method associated with flowering time. Another significant SNP, NC_035437.2-108607891, was also detected related to plant height and flowering time when the MLM method was utilized. Similarly, three significant SNPs (NC_035448.2-178302291, NC_035448.2-178302300, and NC_035448.2-178302303) and two significant SNPs (NC_035445.2-16635243 and NC_035447.2-123860647) were found to be shared by the MLM and the farmCPU methods, respectively. The shared significant SNP, NC_035448.2-178302303, related to seed and plant height detected in the MLM method, was also identified as associated with flowering time by the farmCPU method. In addition, the significant SNPs, NC_035433.2-148595823 [kernel weight (farmCPU), seed weight (farmCPU), and flowering time (farmCPU)], NC_035443.2-174467720 [seed weight (MLM) and plant height (farmCPU-MLM], and NC_035446.2-130491893 [seed weight (farmCPU and flowering time (farmCPU)], were shared. We may conclude that those phenotypes are somehow related to each other, as shown in the correlation test based on the phenotypic data.

Previous studies have highlighted the importance of flowering time in sunflower production, with numerous quantitative trait loci (QTLs) identified across different chromosomes [57,58,59,60,61,62,63,64]. Our GWAS analyses corroborate these findings, revealing significant associations between specific genomic regions and flowering time variation. Identifying candidate genes proximal to these loci provides valuable insights into the molecular mechanisms governing this critical trait.

Plant height and stem diameter are essential to sunflower architecture, influencing agronomic performance and mechanical stability. Previous studies have elucidated the genetic basis of these traits [57,59,60,64], revealing a complex network of QTLs distributed throughout the sunflower genome. Our GWAS expands upon these findings by uncovering novel genomic regions associated with variations in plant height and stem diameter. Identifying candidate genes linked to these traits provides valuable insights into the underlying genetic pathways, offering potential targets for breeding programs to optimize plant architecture for improved resource utilization and yield stability.

Similarly, studies focusing on plant height and stem diameter have elucidated the genetic basis of these traits, uncovering QTLs distributed across the sunflower genome [57,59,60,64]. Our GWAS expands upon these findings by pinpointing additional genomic regions associated with plant height and stem diameter variation. Identifying candidate genes linked to these traits enhances our understanding of the underlying genetic pathways and potential targets for breeding programs to optimize plant architecture.

Seed weight and kernel weight are critical determinants of sunflower yield, with previous studies highlighting the genetic complexity underlying these traits [57,59,60,64]. Our GWAS analyses reveal novel associations between specific genomic regions and seed weight/kernel weight variation, providing valuable insights into the genetic control of these economically important traits. Identifying candidate genes associated with seed development and nutrient accumulation offers opportunities for targeted breeding efforts to enhance yield potential and nutritional quality.

The identified SNPs associated with flowering time, plant height, stem diameter, seed weight, and kernel weight were closely located in several protein-coding genes. The shared SNP ‘NC_035434.2-129219774’ associated with flowering time was detected by both methods. This SNP was closely situated to the gene ‘LOC110920267’ functioning in coding probable membrane-associated kinase regulator 2 functioning in regulating plant development [65]. The SNP ‘NC_035443.2-174467720’, identified by both methods and associated with the plant height, was placed close to the gene ‘LOC110888776’ that codes BAHD acyltransferase BIA1 acting in the stimulation of the flavonoid biosynthesis, acylating primary and specific secondary metabolites, and involving in plant growth [66,67]. There was no significant SNP shared by MLM and farmCPU methods for the stem diameter trait of sunflowers. However, regarding the MLM method, four genes were found close to the most significant SNP, NC_035442.2-10938079. Among those genes, ‘LOC110883981’ encodes the protein NPGR2, identified as a calmodulin-binding protein acting in pollen germination [68]. Similarly, the most significant SNP, NC_035434.2-127341479, detected by farmCPU and associated with stem diameter, was located close to five genes. One of those genes, LOC110921587, enabled hydrolase activity and carbohydrate binding [69]. For the seed weight, the SNP ‘NC_035448.2-178302291’ was the most important one regarding the MLM method, which was close to five genes. Two of those genes, LOC110916982 and LOC110918080, encoded NAC domain-containing protein 75-like, controlling abiotic and biotic stress reactions in plants [70] and heterogeneous nuclear ribonucleoprotein Q, acting in the response of the plant immune system [71], respectively. When we compared the results of the MLM and farmCPU methods, we found that three significant SNPs, NC_035433.2-12278968, NC_035441.2-99824358, and NC_035442.2-73212433, on chromosomes 1, 9, and 10, were shared by both models. Among these genes, the gene ‘LOC110863868’ was functioning in coding cationic amino acid transporter 1, which is an amino acid transporter that plays a significant role in nitrogen distribution throughout the plant, which is necessary to maintain development and growth [72]. As our research findings indicate, we identified essential SNPs closely located to the genes, which play significant roles in plant development, influencing sunflower yield and regulating some other activities.

### 4.1. Heritability Estimate of the Traits

Success in the breeding process largely depends on heritability, a proportion of genetic variance in total phenotypic variance. After two years of data analysis, we assessed the broad-sense heritability of the five characteristics. For flowering time, plant height, stem diameter, seed weight, and kernel weight, the estimated *H*^2^ values were 0.94, 0.89, 0.88, 0.63, and 0.61, respectively. The results indicate that most phenotypic expression for the studied traits is influenced by genetic factors, which could be a good indicator of the selection success to be used in the breeding programs. The studies on heritability estimates also reported similar results on those traits. Many studies have reported that flowering time in sunflowers is highly heritable, with high values of *H*^2^. Supporting the result of our research, Farooq et al. (2021) [73] detected an *H*^2^ of 0.98, and Memon et al. (2014) [74] found it to be 0.67–0.88. For the plant height, Sridhar et al. (2006) [75], Milan et al. (2013) [76], and Komel & Razzaq (2019) [77] estimated high values of *H*^2^ (>0.70), which entirely support our result. Similarly, the heritability estimates for the stem diameter and seed weight that we report in this study were also supported by several reports [73,78,79,80].

### 4.2. Integration of Genomic Knowledge into Breeding Programs

Our study fills a notable gap in sunflower research by leveraging the power of GWAS to decipher the inheritance of complex traits. By integrating advanced genomic methods, we are improving our understanding of sunflower genetics and laying the foundation for more effective breeding strategies. Incorporating GWAS insights into breeding programs promises to develop sunflower varieties tailored to specific environmental conditions, thereby increasing yield potential and resilience to biotic and abiotic stresses. In addition, functional validation of the identified candidate genes and exploration of genotype-environment interactions represent important avenues for future research to enable the development of high-performance sunflower cultivars capable of meeting the changing challenges of global agriculture.

## 5. Conclusions

In summary, our study contributes to sunflower genetics and breeding. Through GWAS, we have deepened our understanding of the genetic determinants underlying vital agronomic traits, paving the way for improved sunflower varieties with higher yield potential, better nutritional quality, and greater resilience to environmental stresses. Future research efforts should focus on translating these genomic insights into concrete breeding outcomes to support global efforts to achieve food security and promote sustainable agricultural practices in sunflower cultivation.

## Data Availability

The genotype data is available in the section “Genotype_data” at https://github.com/ydelen2/Sunflower_important_traits/tree/main/Genotype_data.

## Supplementary Materials

The following supporting information can be downloaded at: https://github.com/ydelen2/SigSNPs_Genes, Table S1: Significant SNPs detected by MLM; Table S2: Significant SNPs identified by farmCPU; Table S3: Gene Annotation for MLM; Table S4: Gene Annotation for farmCPU.

## Appendix A

**Table A1:** Pairwise *p*-values for the Six Sunflower Traits Evaluated.

	PH	FT	HD	SD	SW	KW
**PH**	0	2.77 × 10^−25^	1.59 × 10^−30^	1.55 × 10^−63^	3.23 × 10^−15^	9.95 × 10^−10^
**FT**	3.08 × 10^−25^	0	0.060962	2.67 × 10^−14^	0.101359	0.777435
**HD**	1.33 × 10^−31^	0.020321	0	2.53 × 10^−62^	8.43 × 10^−29^	2.02 × 10^−27^
**SD**	1.11 × 10^−64^	5.35 × 10^−15^	1.94 × 10^−63^	0	1.14 × 10^−22^	1.12 × 10^−16^
**SW**	5.39 × 10^−16^	0.05068	7.66 × 10^−30^	1.43 × 10^−23^	0	3.53 × 10^−97^
**KW**	2.49 × 10^−10^	0.777435	2.02 × 10^−28^	1.60 × 10^−17^	2.35 × 10^−98^	0
